# Potential role of aquaporin 3 in gastric intestinal metaplasia

**DOI:** 10.18632/oncotarget.5370

**Published:** 2015-10-16

**Authors:** Haijian Zhao, Xiaojun Yang, Yangchun Zhou, Weiming Zhang, Yao Wang, Jianfei Wen, Zhihong Zhang, Lizong Shen

**Affiliations:** ^1^ Division of Gastrointestinal Surgery, Department of General Surgery, First Affiliated Hospital, Nanjing Medical University, Nanjing 210029, Jiangsu, China; ^2^ Department of Gastrointestinal Surgery, Huai'an Hospital Affiliated to Xuzhou Medical College, Second People's Hospital of Huai'an City, Huai'an 223002, Jiangsu, China; ^3^ Department of General Surgery, Second Affiliated Hospital, Nanjing Medical University, Nanjing 210011, Jiangsu, China; ^4^ Department of General Surgery, Affiliated Mingde Hospital, Nanjing Medical University, Nanjing 211166, Jiangsu, China; ^5^ Department of Pathology, First Affiliated Hospital, Nanjing Medical University, Nanjing 210029, Jiangsu, China

**Keywords:** gastric intestinal metaplasia, aquaporin 3, gastric cancer, pathology

## Abstract

Gastric intestinal metaplasia (GIM) is a pre-cancerous condition and a pivotal step in the formation of gastric cancer (GC). Aquaporin 3 (AQP3) has been found to be expressed in goblet cells rather than mucus-secreting glands. To investigate the characteristics of GIM in non-cancerous tissues adjacent to GC, as well as the expression and role of AQP3 in GIM tissues, 16 patients diagnosed with gastric adenocarcinoma of intestinal type located in the lesser curve of the antrum were consecutively enrolled in this study. A new pathological technology called “gastric mucosal sausage roll” was introduced. GIM was determined according to the updated Sydney system, and AQP3 expression in goblet cells was determined by immunohistochemistry. GIM was found in all stomach specimens, and its incidence increased with progression to GC (*P* < 0.001). GIM prevalence displayed remarkable association with the distance to GC in the anterior gastric wall tissues (*P* = 0.016) and tissues toward the cardia (*P* = 0.014), such that GIM was more common in the areas closer to GC (*P* < 0.001). AQP3 was found to be expressed in 67.71% of parts with GIM, and AQP3 immunoreactivity was identified more frequently in severe GIM areas (*P* < 0.001). In short, the incidence and severity of GIM correlated with the distance from GC, and AQP3 was differentially expressed in goblet cells, with most AQP3-positive goblet cells presenting in severe GIM. Together, this study suggests that AQP3 may play an important role in gastric carcinogenesis from GIM.

## INTRODUCTION

Although the incidence of gastric cancer (GC) has decreased over the past decades, it remains one of the most common cancers and the third leading cause of cancer-related mortality worldwide [1]. The 5-year overall survival rate of early GC is higher than 90% [2, 3], which drops to 25% or less when the disease is advanced [4, 5]. The pathogenesis of GC remains elusive. However, it is generally agreed upon that the development from normal gastric mucosa to gastric carcinoma is a multistep progression that includes chronic gastritis, chronic atrophic gastritis, intestinal metaplasia, dysplasia and invasive carcinoma [6, 7]. Gastric intestinal metaplasia (GIM), derived mainly from gastric antrum [8], is recognized as a pre-cancerous lesion of GC and a pivotal point in gastric carcinogenesis [9, 10], with GIM patients eventually progressing into intestinal type gastric carcinoma [7, 11–13]. Previous studies that defined the relationship between GIM and GC were mostly based on the long-term follow-up of a cohort of patients by histological examination of gastric biopsies obtained under upper endoscopy, yet the pathogenesis of GIM in gastric carcinogenesis is still unclear [14–17].

Aquaporins (AQPs) are a family of small, integral membrane proteins that transport water and, in some cases, water and glycerol [18, 19]. Mounting evidence further implicates the role of AQPs in promoting cancer cell migration and proliferation, adding AQPs to an expanding list of effectors in tumor biology [20]. Previously, we have demonstrated that AQP3 is overexpressed in GC, and that its expression is associated with histological classification, lymph node metastasis and lymphovascular invasion [21]. Furthermore, we showed that upregulation of AQP3 can promote the migration and proliferation of human gastric cancer cells via promoting epithelial-to-mesenchymal transition, indicating that AQP3 plays an important role in gastric cancer carcinogenesis and progression [22–24].

In our previous study (data not shown), AQP3 was found to be expressed in goblet cells rather than mucus-secreting glands in the para-tumor non-cancerous gastric mucosa tissues. However, the role of AQP3 in GIM remains unclear. In the present study, we investigated the incidence and severity of GIM in the non-cancerous gastric mucosa tissues adjacent to GC, as well as the expression of AQP3 in these tissues. Additionally, we evaluated the correlation of GIM with GC, along with the cross-correlation with AQP3 expression. We found that GIM was a common event in the non-cancerous gastric mucosa tissues adjacent to GC, and its incidence and severity positively correlated with the distance from GC. Specifically, AQP3 was expressed in most GIM, and AQP3 expression was associated with severe GIM. These preliminary findings improve our understanding of the pathogenesis of GIM in gastric carcinogenesis, and indicate a potential role of AQP3 in the development from GIM to GC.

## RESULTS

### Incidence and severity of GIM correlates with distance from GC

Sixty-four gastric mucosal sausage rolls were obtained from 16 GC patients, and 192 parts were evaluated for severity of GIM. GIM was found in all stomach specimens, and 50% of the 192 parts exhibited GIM, with mild, moderate and severe GIM being detected in 7.8% (15/192), 14.6% (28/192) and 27.6% (53/192) parts respectively. As shown in Table 1, the incidence of GIM was 68.8% (44/64), 48.4% (31/64) and 32.8% (21/64) in parts A, B and C respectively (χ^2^ = 25.960, *P* < 0.001). There was no significant difference in the incidence of GIM with regard to the distance to GC in the posterior gastric wall tissues (χ^2^ = 5.586, *P* = 0.471) and tissues toward the pylorus (χ^2^ = 3.359, *P* = 0.763). However, GIM prevalence displayed remarkable association with the distance to GC in the anterior gastric wall tissues (χ^2^ = 15.571, *P* = 0.016) and tissues toward the cardia (χ^2^ = 15.921, *P* = 0.014). Regarding the severity of GIM, marked GIM was more common in the areas adjacent to GC, and the incidence of marked GIM was 48.4% (31/64), 20.3% (13/64) and 14.0% (9/64) in part A, B and C respectively (χ^2^ = 21.475, *P* < 0.001). These results indicated that the incidence and severity of GIM was associated with the distance to GC. Furthermore, GIM became more common and more severe with proximity to GC lesions, which strongly suggested an association between GIM and gastric carcinogenesis.

**Table 1A:** Correlation between the incidence or severity of GIM and the distance from GC (cases, %)

	Grade of GIM				χ^2^	*P*
	0	1	2	3		
A	20 (31.25)	5 (7.81)	8 (12. 5)	31 (48.44)	25.960	<0.001
B	33 (51.56)	7 (10.94)	11 (17.19)	13 (20.31)		
C	43 (67.19)	3 (4.69)	9 (14.06)	9 (14.06)		

**Table 1B:** Correlation between the incidence or severity of GIM and the distance from GC (cases, %) according to different direction

	Grade of GIM				χ^2^	*P*
	0	1	2	3		
3 o'clock (posterior wall)					5.586	0.471
A	9 (56.25)	0 (0.00)	1 (6.25)	6 (37.50)		
B	8 (50.00)	2 (12.50)	3 (18.75)	3 (18.75)		
C	10 (62.50)	2 (12.50)	2 (12.50)	2 (12.50)		
6 o'clock (pylorus direction)					3.359	0.763
A	4 (25.00)	2 (12.50)	4 (25.00)	6 (37.50)		
B	8 (50.00)	1 (6.25)	3 (18.75)	4 (25.00)		
C	8 (50.00)	1 (6.25)	4 (25.00)	3 (18.75)		
9 o'clock (anterior wall)					15.571	0.016
A	4 (25.00)	2 (12.50)	1 (6.25)	9 (56.25)		
B	10 (62.50)	1 (6.25)	2 (12.50)	3 (18.75)		
C	14 (87.50)	0 (0.00)	0 (0.00)	2 (12.50)		
12 o'clock (cardiac direction)					15.921	0.014
A	3 (18.75)	1 (6.25)	2 (12.50)	10 (62.50)		
B	7 (43.75)	3 (18.75)	3 (18.75)	3 (18.75)		
C	11 (68.75)	0 (0.00)	3 (18.75)	2 (12.50)		

### AQP3 may be involved in carcinogenesis from GIM to GC

**Figure 1:**
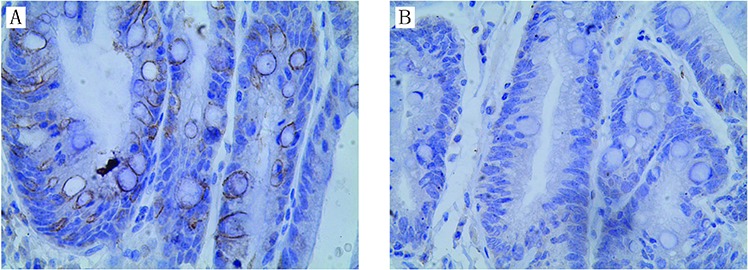
Expression of AQP3 in gastric intestinal metaplasia (GIM) Strong AQP3 immunoreactivity was identified in GIM **A.** negative AQP3 in GIM **B.** Original magnification, × 400.

As indicated in the previous experiments, AQP3 may be expressed in GIM rather than mucus-secreting glands. In this study, AQP3 was found to be expressed in 67.71% (65/96) of the parts with GIM (Figure 1A), and it was absent in 32.29% (31/96) (Figure 1B). Consistent with our previous reports [21, 24], AQP3 expression increased in all GC tissues.

As shown in Table 2A, AQP3 immunoreactivity was not found in mild GIM areas, and it was identified more frequently in severe GIM areas (χ^2^ = 40.280, *P* < 0.001). We further investigated the difference between the percentage of AQP3 positive goblet cells in grade 2 and 3 GIM, and more AQP3 positive cells were found in grade 3 GIM than that in grade 2 GIM (Table 2B, χ^2^ = 6.720, *P* = 0.01). These results indicated that AQP3 immunoreactivity in goblet cells was in consistent with the severity of GIM. However, a significant relationship between AQP3 immunoreactivity and distance from GC was not found (Table 3, χ^2^ = 1.958, *P* = 0.376).

**Table 2A:** Correlation between AQP3 expression with the grade of GIM

AQP3 immunoactivity	GIM grade				χ^2^	*P*
	0	1	2	3		
positive	0	0	19	46	40.280	<0.001
negative	0	15	9	7		

**Table 2B:** The different percentage of AQP3 positive goblet cells between the grade 2 and 3 GIM

percentage of AQP3 positive goblet cells	GIM grade		χ^2^	*P*
	2	3		
25–50%	8	6	6.720	0.01
>50%	11	40		

**Table 3:** Relationship of AQP3 immunoreactivity to the distance from GC

AQP3 immunoactivity	A	B	C	χ^2^	*P*
positive	32	18	15	1.958	0.376
negative	12	13	6		

## DISCUSSION

GIM is recognized as a precancerous lesion of gastric carcinoma [9, 10]. However, it is very difficult to determine its precise association with GC, and the pathogenesis of GIM in gastric carcinogenesis needs to be elucidated further. In the present study, we introduced a new pathological technology, called the “gastric mucosal sausage roll”. This method helped us to investigate the overall distribution of GIM in non-cancerous tissues around GC. GIM has been revealed to mainly derive from the gastric antrum [8], and is associated with gastric carcinoma of intestinal type [7, 11–13]. Therefore, for this retrospective study, only patients with gastric adenocarcinoma of intestinal type located in the lesser curve of the antrum were enrolled, and the design helped us to study the distribution pattern of GIM.

In this study, we reveal that GIM is a common event in the non-cancerous tissues around GC. However, we demonstrate for the first time that the occurrence and severity of GIM in different parts of the stomach around GC was not uniform. As a whole, the incidence of GIM was significantly more common in the areas adjacent to GC than in the distant areas. Importantly, the prevalence of GIM displayed a remarkable association with the distance to GC in the anterior gastric wall tissues and in the tissues toward the cardia, which was consistent with the GIM prevalence in different parts of the stomach from de Vries et al.′s study [25]. However, these associations were not found in the posterior gastric wall tissues or the tissues toward the pylorus. More importantly, the severity of GIM was positively associated with the distance from GC. Although we do not know the mechanism for the differential prevalence in different directions around GC, these findings ascertained that GIM becomes more severe as it nears GC lesions. Our study presents direct pathologic evidence of GIM in the development of intestinal type gastric carcinoma, compared with the results from long-term follow-up of GIM patients [14–17], which was in accordance with that of an Italian study [26] that showed that the risk of gastric neoplasms increased with increasing GIM extension, especially in patients with GIM extension 20% or more. Thus, it may be inferred that GIM is a key process in the carcinogenesis of GC.

How gastric GIM progresses to gastric carcinoma remains unclear. Some proteins have been demonstrated to be differentially expressed in GIM, such as high expression of CD24 and leucine-rich repeat-containing G protein-coupled receptor 5 (LGR5), positive expression of Das-1 and Ki-67, and low expression or loss of Gastrokine-2 (GKN-2) [27–30], but their roles in GIM and malignant transformation are unknown. Our previous studies have established a role for AQP3 in gastric carcinogenesis and gastric carcinoma progression [21–24]. This retrospective study confirmed our previous serendipitous finding that AQP3 is expressed distinctively in goblet cells rather than mucus-secreting glands in the non-cancerous gastric mucosa tissues, which sparked our interest to perform this study. Most importantly, the association of AQP3 immunoreactivity with severe GIM was identified in this study.

AQP3 has been found to be upregulated in gastric carcinoma tissues [21, 24]. Moreover, AQP3 is expressed differentially in goblet cells, with most of the AQP3-positive goblet cells presenting in severe GIM, and the severity of GIM was positively associated with the distance from GC. Collectively, this leads us to speculate that AQP3 plays a potential role in gastric carcinogenesis that develops from GIM, possibly through inflammatory carcinoma transformation in the stomach.

In part, because of a lack of convincing indicators for predicting the risk of transformation from GIM to GC, there is generally no recognized consensus on how to perform a follow-up for GIM. The European guidelines on the management of precancerous conditions and lesions in the stomach (MAPS) recommend that every 3 years there be an endoscopic follow-up uniformly for all GIM patients [8]. However, this uniform approach does not allow for a patient-tailored approach and potentially exposes patients, most of which would never develop gastric cancer, to unnecessary procedures [31]. Therefore, we propose that AQP3 may be useful for identifying at-risk patients, although this present study is preliminary. In the future, we propose to elucidate the exact role of AQP3 in GIM malignant transformation by performing an animal experiment with *Helicobacter pylori*-induced gastritis or gastric carcinoma models [32], as well as a prospective, randomized, multicenter clinical trial.

## MATERIALS AND METHODS

### Human gastric tissue specimens

Sixteen patients diagnosed with gastric adenocarcinoma of intestinal type located in the lesser curve of the antrum (*n* = 16; median age, 62.25 ± 12.40 years; range, 44–86 years) between September and November 2014 at the Department of General Surgery, First Affiliated Hospital, Nanjing Medical University, were consecutively enrolled in this study. All patients were diagnosed pathologically according to the American Joint Committee on Cancer (AJCC) criteria. None of these patients had received chemotherapy or radiotherapy prior to surgery. All patients underwent surgical treatment with curative intent for GC, defined as performing the surgeries with the goal of achieving R0 resection. The clinical characteristics of these patients are listed in Table 4. No case with distant metastasis was found in this study. Patient samples were obtained following written consent according to an established protocol approved by the Nanjing Medical University Institutional Review Board. This study was also in compliance with the Declaration of Helsinki.

**Table 4:** Clinicopathological features of patients diagnosed with GC in this study

Case No	Age	Sex	Tumor size(cm)	Pathologic T classification	Lymph node metastatis	Differentiation
1	74	F	3.2	T1b	0/17	MD
2	47	M	3.0	T1a	0/20	PD
3	81	F	3.0	T3	0/24	PD
4	47	F	2.3	T1b	0/45	PD
5	68	M	2.0	T2	0/28	MD
6	86	M	3.0	T1a	0/23	WD
7	44	M	1.5	T1a	0/21	WD
8	60	M	3.1	T1b	0/34	PD
9	56	F	2.5	T1b	0/38	WD
10	52	F	0.3	T1a	0/18	MD
11	61	F	2.5	T1a	0/36	MD
12	64	F	1.5	T1a	0/32	WD
13	73	M	1.0	T1a	0/42	WD
14	69	M	2.5	T2	4/46	PD
15	52	F	2.7	T1a	0/40	PD
16	62	F	2.2	T1a	0/25	PD

WD: well differentiated; MD: moderately differentiated; PD: poorly differentiated

**Figure 2:**
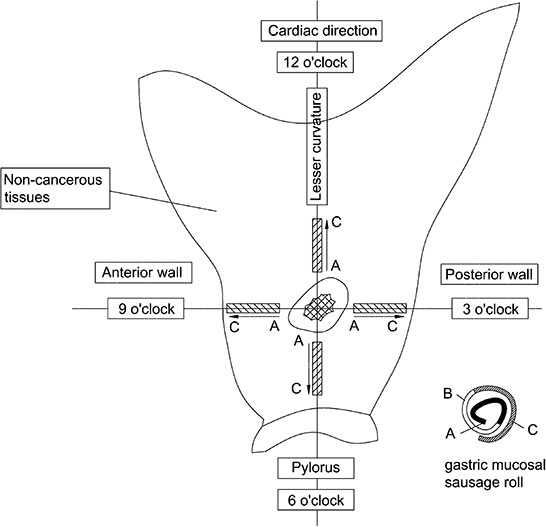
Diagrammatic sketch for “gastric mucosal sausage roll” technique The stomach specimens were cut along the greater curvature, and non-cancerous gastric mucosa tissues adjacent to GC were obtained with widths of 5 mm and lengths > 2 cm, without vascular tissues in the four directions of 3, 6, 9 and 12 o'clock around the GC lesions, which corresponded to the posterior wall, pylorus, anterior wall and cardiac directions, respectively. Each tissue band was rolled like a sausage from the closest end to the farthest end with respect to the GC lesion.

All resected stomach specimens were immediately fixed in 10% phosphate-buffered formalin. At least 12 hours later, the stomach specimens were cut along the greater curvature. As shown in Figure 2, non-cancerous gastric mucosa tissues adjacent to GC were obtained with width 5 mm and length > 2 cm, without vascular tissues in the four directions of 3, 6, 9 and 12 o'clock around the GC lesions, which corresponded to the posterior wall, pylorus, anterior wall and cardiac directions, respectively. Each tissue band was rolled like a sausage from the closest end to the farthest end with respect to GC lesion, which we termed “gastric mucosal sausage roll”. These rolls were embedded in paraffin, and the transaction sections were obtained to evaluate GIM and AQP3 immunoreactivity by two experienced gastrointestinal pathologists that were blinded to the study. Each section was divided into three parts, A (≤1 cm), B (1–2 cm) and C (>2 cm), according to the distance to the margin of GC lesion.

### Identification of gastric intestinal metaplasia

Sections (5-μm-thick) were deparaffinized and stained with hematoxylin and eosin (HE staining). The presence of goblet cells indicated GIM. According to the updated Sydney system [33], GIM were graded as absent, mild, moderate or marked (grades 0–3, respectively). The two pathologists determined the grade of GIM independently.

### Immunohistochemical detection of AQP3 in goblet cells

Expression of AQP3 in goblet cells was determined by immunohistochemistry (IHC), as described previously [34]. A polyclonal rabbit anti-AQP3 antibody was obtained from Santa Cruz Biotechnology (Santa Cruz, CA). Two pathologists scored protein expression as the percentage of positive goblet cells (scale 0–100%) with a staining intensity from 0–3+. Positive IHC expression was defined as > 25% staining with an intensity of 2–3+.

### Statistical analysis

Pearson's chi-square test was used to examine the association between various clinicopathological parameters. Statistical analyses were performed with SPSS version 11.0 (SPSS Inc., Chicago, IL, USA), and *P* < 0.05 was considered statistically significant.

## References

[1] Ferlay J, Soerjomataram I, Dikshit R, Eser S, Mathers C, Rebelo M, Parkin DM, Forman D, Bray F (2015). Cancer incidence and mortality worldwide: sources, methods and major patterns in GLOBOCAN 2012. Int J Cancer.

[2] Maruyama K, Kaminishi M, Hayashi K, Isobe Y, Honda I, Katai H, Arai K, Kodera Y, Nashimoto A (2006). Japanese Gastric Cancer Association Registration Committee: Gastric cancer treated in 1991 in Japan: data analysis of nationwide registry. Gastric Cancer.

[3] Shen L, Huang Y, Sun M, Xu H, Wei W, Wu W (2009). Clinicopathological features associated with lymph node metastasis in early gastric cancer: analysis of a single-institution experience in China. Can J Gastroenterol.

[4] Jemal A, Siegel R, Ward E, Hao Y, Xu J, Murray T, Thun MJ (2008). Cancer statistics. CA Cancer J Clin.

[5] Hartgrink HH, Jansen EP, van Grieken NC, van de Velde CJ (2009). Gastric cancer. Lancet.

[6] Correa P (1988). A human model of gastric carcinogenesis. Cancer Res.

[7] Correa P (1992). Human gastric carcinogenesis: a multistep and multifactorial process-first American Cancer Society Award Lecture on Cancer Epidemiology and Prevention. Cancer Res.

[8] Dinis-Ribeiro M, Areia M, de Vries AC, Marcos-Pinto R, Monteiro-Soares M, O'Connor A, Pereira C, Pimentel-Nunes P, Correia R, Ensari A, Dumonceau JM, Machado JC, Macedo G (2012). Management of precancerous conditions and lesions in the stomach (MAPS): guideline from the European Society of Gastrointestinal Endoscopy (ESGE), European Helicobacter Study Group (EHSG), European society of Pathology (ESP), and the Sociedade Portuguesa de Endoscopia Digestiva (SPED). Endoscopy.

[9] de Vries AC, Kuipers EJ (2007). Epidemiology of premalignant gastric lesions: implications for the development of screening and surveillance strategies. Helicobacter.

[10] Zullo A, Hassan C, Romiti A, Giusto M, Guerriero C, Lorenzetti R, Campo SM, Tomao S (2012). Follow-up of intestinal metaplasia in the stomach: When, how and why. World J Gastrointest Oncol.

[11] Bronner MP (1999). Gastric cancer and intestinal metaplasia. Hum Pathol.

[12] Rauws EA, Langenberg W, Houthoff HJ, Zanen HC, Tytgat GN (1988). Campylobacter pyloridis-associated chronic active antral gastritis. A prospective study of its prevalence and the effects of antibacterial and antiulcer treatment. Gastroenterology.

[13] Correa P (1982). Precursors of gastric and esophageal cancer. Cancer.

[14] Uemura N, Okamoto S, Yamamoto S, Matsumura N, Yamaguchi S, Yamakido M, Taniyama K, Sasaki N, Schlemper RJ (2001). Helicobacter pylori infection and the development of gastric cancer. N Engl J Med.

[15] de Vries AC, Kuipers EJ (2007). Epidemiology of premalignant gastric lesions: implications for the development of screening and surveillance strategies. Helicobacter.

[16] Cho SJ, Choi IJ, Kim CG, Kook MC, Lee JY, Kim BC, Ryu KH, Nam SY, Kim YW (2010). Risk factors associated with gastric cancer in patients with a duodenal ulcer. Helicobacter.

[17] de Vries AC, van Grieken NC, Looman CW, Casparie MK, de Vries E, Meijer GA, Kuipers EJ (2008). Gastric cancer risk in patients with premalignant gastric lesions: a nationwide cohort study in the Netherlands. Gastroenterology.

[18] Agre P (2006). The aquaporin water channels. Proc Am Thorac Soc.

[19] Magni F, Sarto C, Ticozzi D, Soldi M, Bosso N, Mocarelli P, Kienle MG (2006). Proteomic knowledge of human aquaporins. Proteomics.

[20] Hu J, Verkman AS (2006). Increased migration and metastatic potential of tumor cells expressing aquaporin water channels. FASEB J.

[21] Shen L, Zhu Z, Huang Y, Shu Y, Sun M, Xu H, Zhang G, Guo R, Wei W, Wu W (2010). Expression profile of multiple aquaporins in human gastric carcinoma and its clinical significance. Biomed Pharmacother.

[22] Chen J, Wang T, Zhou YC, Gao F, Zhang ZH, Xu H, Wang SL, Shen LZ (2014). Aquaporin 3 promotes epithelial-mesenchymal transition in gastric cancer. J Exp Clin Cancer Res.

[23] Wang J, Gui Z, Deng L, Sun M, Guo R, Zhang W, Shen L (2012). c-Met upregulates aquaporin 3 expression in human gastric carcinoma cells via the ERK signalling pathway. Cancer Lett.

[24] Huang Y, Zhu Z, Sun M, Wang J, Guo R, Shen L, Wu W (2010). Critical role of Aquaporin-3 in the human epidermal growth factor-induced migration and proliferation in the human gastric adenocarcinoma cells. Cancer Biol Ther.

[25] de Vries AC, Haringsma J, de Vries RA, Ter Borg F, van Grieken NC, Meijer GA, van Dekken H, Kuipers EJ (2010). Biopsy strategies for endoscopic surveillance of pre-malignant gastric lesions. Helicobacter.

[26] Tava F, Luinetti O, Ghigna MR, Alvisi C, Perego M, Trespi E, Klersy C, Fratti C, Fiocca R, Solcia E (2006). Type or extension of intestinal metaplasia and immature/atypical “indefinite-for-dysplasia” lesions as predictors of gastric neoplasia. Hum Pathol.

[27] Wang YC, Wang JL, Kong X, Sun TT, Chen HY, Hong J, Fang JY (2014). CD24 mediates gastric carcinogenesis and promotes gastric cancer progression via STAT3 activation. Apoptosis.

[28] Zheng ZX, Sun Y, Bu ZD, Zhang LH, Li ZY, Wu AW, Wu XJ, Wang XH, Cheng XJ, Xing XF, Du H, Ji JF (2013). Intestinal stem cell marker LGR5 expression during gastric carcinogenesis. World J Gastroenterol.

[29] Feng XS, Wang YF, Hao SG, Ru Y, Gao SG, Wang LD (2013). Expression of Das-1, Ki67 and sulfuric proteins in gastric cardia adenocarcinoma and intestinal metaplasia lesions. Exp Ther Med.

[30] Dai J, Zhang N, Wang J, Chen M, Chen J (2014). Gastrokine-2 is downregulated in gastric cancer and its restoration suppresses gastric tumorigenesis and cancer metastasis. Tumour Biol.

[31] Zullo A, Hassan C, Repici A, Annibale B (2013). Intestinal metaplasia surveillance: Searching for the roadmap. World J Gastroenterol.

[32] Wang G, Gao F, Zhang W, Chen J, Wang T, Zhang G, Shen L (2012). Involvement of Aquaporin 3 in Helicobacter pylori-related gastric diseases. PLoS One.

[33] Dixon MF, Genta RM, Yardley JH, Correa P (1996). Classification and grading of gastritis. The updated Sydney System. International Workshop on the Histopathology of Gastritis, Houston 1994. Am J Surg Pathol.

[34] Zhou Y, Wang Y, Wang S, Shen L (2015). Hyperglycemia promotes human gastric carcinoma progression via aquaporin 3. Dig Dis Sci.

